# Supplementary material for: Semiconductor-less vertical transistor with ION/IOFF of 106
Source: Nat Commun. 2021 Feb 12;12:1000. doi: 10.1038/s41467-021-21138-y (PMC7881104; doi:10.1038/s41467-021-21138-y)
Supplement: Supplementary file 1 — Supplementary Information [file 41467_2021_21138_MOESM1_ESM.pdf]

Supplementary Information

Supplementary Note 1: Capacitive Coupling among C_{TC}, C_{Gate}, and C_Q

Here, we demonstrate the limitations of the work function modulation of graphene by the coupling effect among the tunneling-channel capacitance (C_{TC}), gate capacitance (C_{Gate}) and quantum capacitance (C_Q), as shown in Supplementary Fig. 1. Capacitive coupling between C_D and C_G will be addressed, and the effect of C_Q will be added. Assuming that only C_G and C_D are serially connected by a metal electrode in which charge Q accumulates, the potential of the electrode (V_M) is determined as follows:

$$(C_{TC} + C_{Gate})V_M = C_{Gate} V_G + C_{TC} V_D + Q. \tag{1}$$

If $V_G > 0$, $V_D > 0$ and if the middle electrode is replaced by grounded graphene, the accumulated charge consists of electrons and can be expressed as

$$Q = -en = \frac{e}{\pi}\left(\frac{e\varphi_{gr}}{\hbar v_F}\right)^2, \tag{2}$$

where φ_{gr} is the potential difference of graphene from the Dirac point resulting from the charge. If the work function of the gate and drain electrode is assumed to be identical to the Dirac point, for simplicity without loss of generality, V_M can be set as equal to φ_{gr} for charge Q. Therefore, φ_{gr} can be determined as follows:

$$C_{Gate} V_G + C_{TC} V_D = \frac{e}{\pi}\left(\frac{e}{\hbar v_F}\right)^2 \varphi_{gr}{}^2 + (C_{TC} + C_{Gate})\varphi_{gr}. \tag{3}$$

The left term is the fictitious charge (Q_{fic}), which is determined by the device structure (C_{Gate} and C_{TC}) and operating conditions (V_G and V_D); the right term is the quadratic function of φ_{gr}. Therefore, varying the applied voltage of V_G or V_D resulted in a shift of φ_{gr}. Considering the small modulation of drain and gate voltages with φ_{gr} (or I_D) unchanged, the above equation can be

$$C_{Gate}\, dV_G + C_{TC} dV_D = 0. \tag{4}$$

Therefore, the gain of V_D over V_G can be estimated with the ratio of C_{Gate}/C_{TC}. To increase the gain, the thickness of the tunneling channel should increase, and that of the gate dielectric should decrease.

Supplementary Note 2: Array approximation of field emission from graphene near Dirac point.

As shown in Supplementary Fig. 2a, the FE of planar graphene could be treated as an array of numerous (FE current) emitters because electron-hole puddles exist in graphene[1,2]. The FE current was analyzed based on such an array using Seppen-Katamuki (SK, or “intercept-slope” in Japanese) plots[3] by extracting the slope and y-intercept values from the drain current using the axes $\ln(I_D/V_D^2)$ and $1/V_D$. For this analysis, the FE currents of the device measured at 15 K were plotted in Supplementary Fig. 2b. Then, the SK-plots were obtained, as described in Supplementary Fig. 2c.

Unlike the single-emitter assumption, which appears as a single point in the SK-plot, the linear distribution in Supplementary Fig. 2c implies the log-normal distributions of the radii or heights of the emitters[4]. The absolute value of the slope in the SK-plot decreases as the gate bias increases. Since the slope in the SK-plot is proportional to $\Phi_B^{3/2}$ (ref. 5), Φ_B for electron decreases by 8 % as the gate bias increases from 0 V to 12 V. Although the Fermi level of graphene varies according to the presence of impurities and defects[6], we can assume that the Fermi level of our device is at the Dirac point when $V_D = V_G = 0$ V because of the *h*BN-graphene-*h*BN sandwich structure. Then, we obtained that the slope of the SK-plot raised to the power of 2/3 monotonically decreases relative to the charge carrier density raised to the power of 1/2 (Supplementary Fig. 2d). The linear relationship between the slope in the SK-plot and the accumulated charges in the graphene validates the analysis because both are proportional to the work function of graphene

Supplementary Note 3: Improvement of work function modulation by adopting high-k insulators

The engineering of gate insulators is another way to improve the work function modulation of the FEB. High-dielectric constant materials, such as hafnium oxide (HfO_x) and aluminum oxide (AlO_x), can be used to accumulate more charge without breaking the insulator. Such materials have been traditionally used to accumulate an identical charge at a lower voltage[7]. To explore how to improve the switching performance by engineering a gate insulator, we calculated the graphene work function shifts by varying the high-k material and its thickness for C_{Gate}, as shown in Supplementary Fig. 4. The upper left part of the graph shows the work function shift with an HfO_x gate dielectric; the lower right part of the graph shows the work function shift with either *h*BN or SiO_2. In both cases, *h*BN was used as the tunneling channel. We fixed V_D at half of the breakdown voltage of the tunneling channel and changed V_G from 0 V to half of the breakdown voltage of the gate insulator. Although the calculated work function shift was 0.14 eV for SiO_2, the shift became 0.5 eV or higher for HfO_x[8]. Thus, the work function shift of FEB can be increased using high-k gate materials.

Supplementary Note 4: Poole-Frenkel Transport through defects of *h*BN

Poole-Frenkel (PF) Transport is a thermally assisted electron emission under high electric field, mediated by defect states in insulators. The current depends on temperature such as $\ln\frac{I_{PF}}{V_D} \propto \left(U_0 - \sqrt{\frac{eV_D}{\varepsilon_r\varepsilon_0}}\right)\frac{1}{K_BT}$, where I_{PF} is Poole-Frenkel current, V_D is drain voltage, U_0 is energy depth from conduction band minimum to defect level of the insulator, ε_r is a dielectric constant of the insulator, ε_0 is vacuum permittivity, K_B is Boltzmann constant, and T is temperature. The I_D of FEB was measured at the temperatures of 78, 115, 175, 225 and 275 K, by varying V_G. It depends on temperature under very high electric field, as shown in Supplementary Fig. 5a.

To investigate the transport mechanism, we extracted the U_0 of each V_G from the above equation. We observed that $\ln(I_D/V_D)$ linearly depended on $1/k_BT$, and U_0 was extracted to around 0.95 eV for all V_G. Interestingly, the minimum temperature, where PF transport dominates, increases as V_G increases (Supplementary Fig. 5b). When V_G was lower than 4 V, I_D was governed by the PF Process at 125 K and higher temperature. As V_G increases (Fermi-level of graphene increases), the minimum temperature of the PF process also increases. It is because FE current becomes dominant when the energy of graphene's electron becomes more significant than that of the thermally excited electron in the defect. Therefore, we conclude that the temperature-dependent current of FEB is originated from PF transport, mediated by defects in *h*BN.

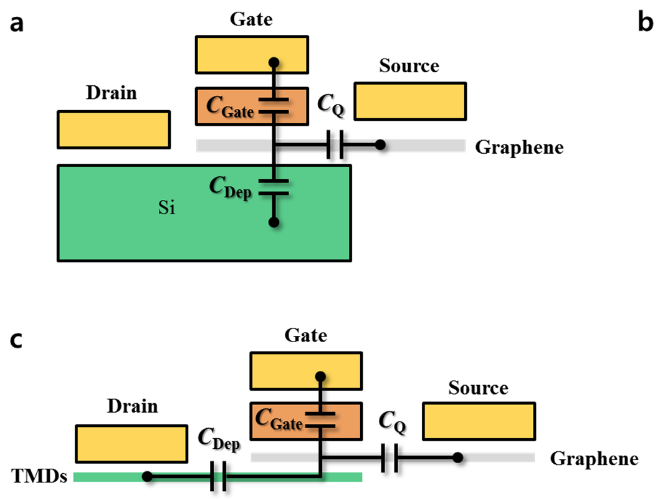

Supplementary Figure 1 Capacitive coupling of various novel devices: **a**, GB, **b**, FEtT, and **c**, GB with TMDs

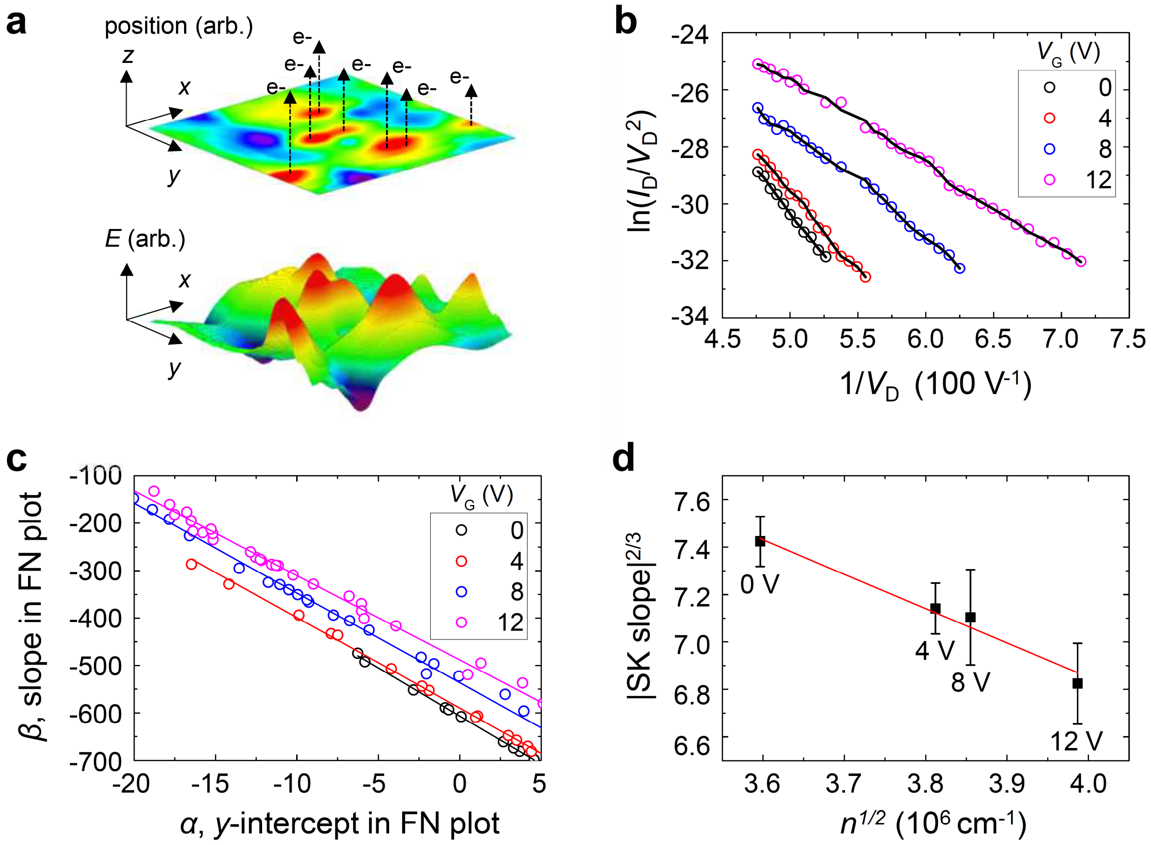

Supplementary Figure 2 Analysis of FE using an array of numerous field emitters. **a**, Field emitter array of planar graphene with electron-hole puddles, where x, y and z axes are positions in the arbitrary unit and E is the energy of an electron. Each electron puddle can be an emission center because it corresponds to a local maximum of the electron energy, *i.e.* a local minimum of the work function. **b**, The output characteristic of the FEB composed of monolayer graphene, gate-*h*BN (thickness : 42.8 nm), and tunneling-*h*BN (thickness : 30.7 nm). This graph exhibits a linear relationship with the deviations on axes of ln(I_D/V_D^2) and 1/V_D. The linear relationship confirms the FE current; the deviation originates from the array of field emitters. **c**, SK-plot of the FE of FEBs. The linear relationship suggests that the array of emitters has an identical work function, and its shift with V_G suggests that the work function is modulated. **d**, Work function

modulation according to the square root of the charge. The linear relationship confirms that the work function shift of graphene originates from the accumulated charge on the graphene, where the charge is extracted from V_G.

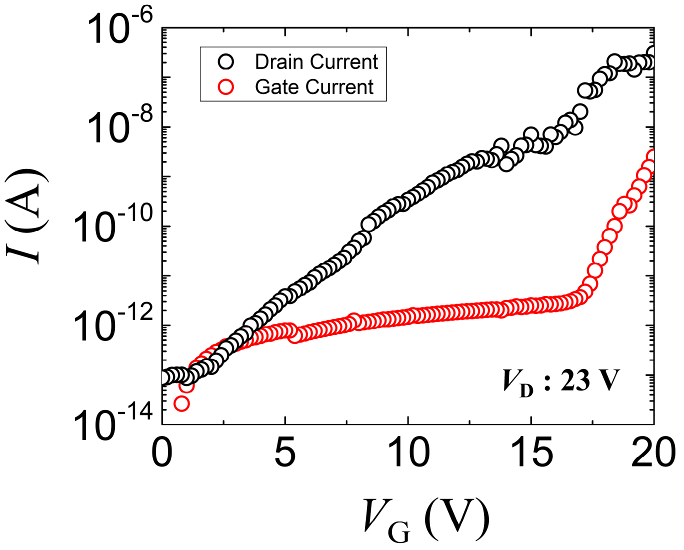

Supplementary Figure 3 Variation of drain and gate current with gate voltage for FEB. The device is composed of monolayer graphene, gate-*h*BN (thickness : 54.4 nm), and tunneling-*h*BN (thickness : 54.8 nm). In the FEB, Both of drain and gate current increase with gate voltage because the gate voltage affects them in different ways. In case of the drain current, it depends on modulation of tunneling barrier height caused by charge accumulation on graphene under the gate voltage and instantly increases with the gate voltage. In contrast, the gate current is mainly affected by electric field applied to the gate-*h*BN, and this makes the gate current start to increase at threshold gate voltage where the gate current is converted from DT to FE. Therefore, when the gate voltage was applied to 20 V near its threshold (17 V), the drain current was 120 times higher than the gate current as shown in Supplementary Fig. 3.

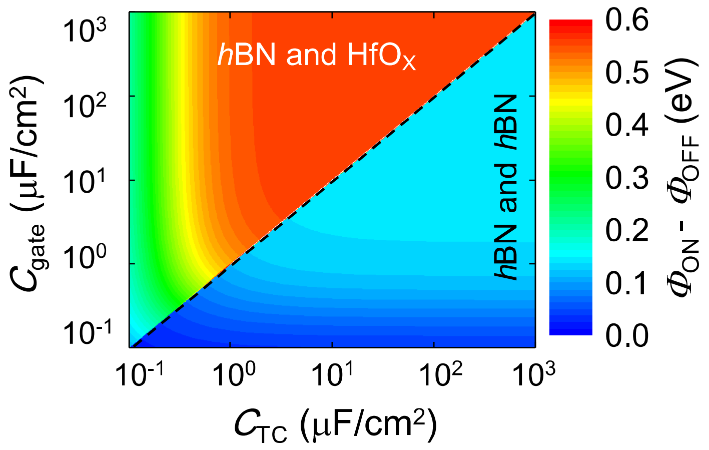

Supplementary Figure 4 Color mapping of the work function shift of FEB as a function of C_{gate} and C_{TC}. The work function shift was calculated in different thickness of *h*BN (lower right) or hafnium oxide (upper left) as gate dielectric, when V_{D} is applied half of breakdown voltage of tunneling channel and V_{G} spans from 0 V to half of the breakdown voltage of the gate dielectric.

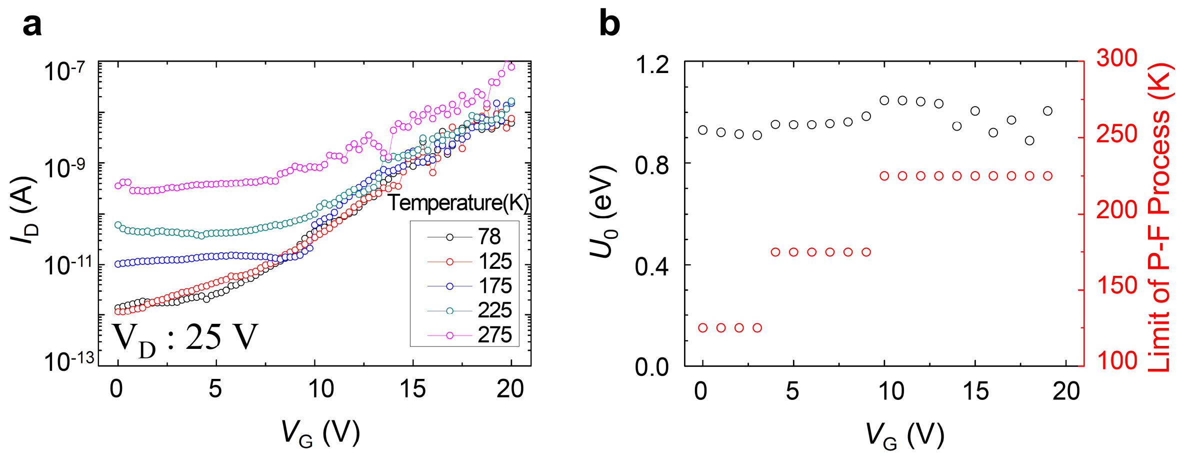

Supplementary Figure 5 Poole-Frenkel Transport through defects of *h*BN. **a**, Transfer characteristic curves were measured with the temperature range of 78–275 K. The I_D of FEB under very high electric field increases by 2 orders, as the temperature increases. **b**, U_0 was extracted for each V_G using the Poole-Frenkel model, and the minimum temperature, where the transport was applicable, was also obtained. For the former, the extracted U_0 was around 0.97 eV for all V_G. For the latter, the minimum temperature increases with V_G; with V_G less than 4 V, PF model applied to the temperature higher than 125 K; with V_G greater than 10 V, the minimum temperature increased to 225 K.

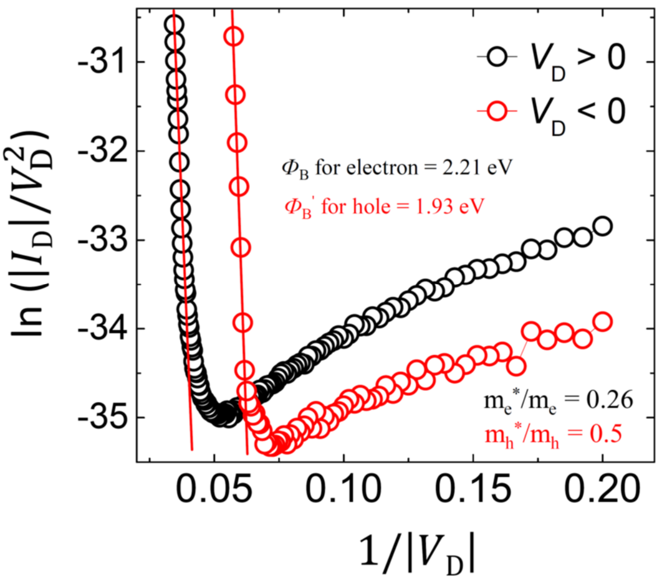

Supplementary Figure 6 Fowler-Nordheim (FN) plot behavior of FEB. The IV curves of Fig. 1h, and i (15 K, V_G=0) were replotted with axes of ln ($|I|/V^2$) and $1/|V|$. The barrier height for the electron (Φ_B) and hole (Φ_B') were extracted from each slope of a line fitted to FN plot and estimated to be 2.21 eV and 1.93 eV, respectively.

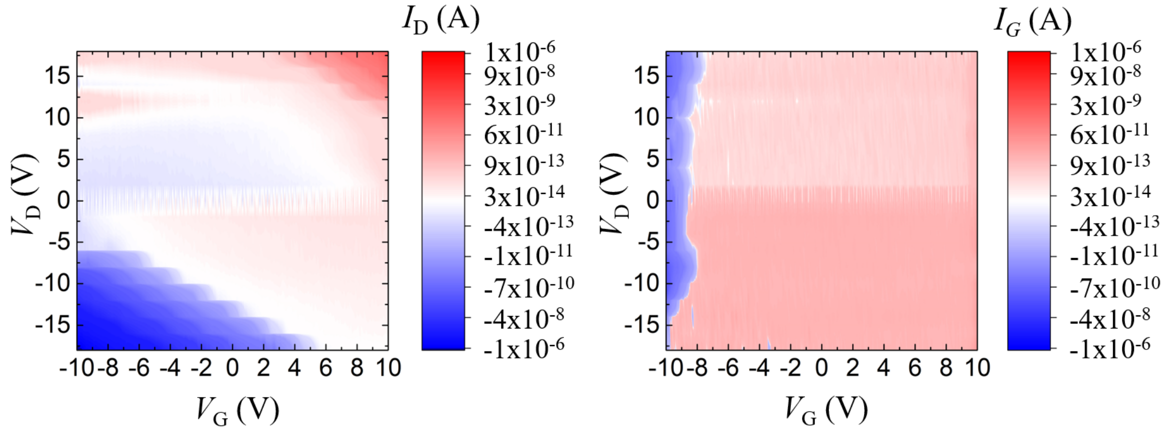

Supplementary Figure 7 Color mapping of the I_D and I_G of FEB device with channel-*h*BN 47 nm and gate-*h*BN 41 nm, as a function of V_D and V_G in a linear scale. Electron current is modulated under positive V_D, and hole current is under negative V_D.

Supplementary References

1. Xue, J. *et al.* Scanning tunnelling microscopy and spectroscopy of ultra-flat graphene on hexagonal boron nitride. *Nat. Mater.* **10**, 282-285 (2011)

2. Martin, J. *et al.* Observation of electron-hole puddles in graphene using a scanning single-electron transistor. *Nat. Phys.* **4**, 144-148

3. Ishikawa, J. *et al.* Estimation of metal-deposited field emitters for the micro vacuum tube. *Jpn. J. Appl. Phys.* **32**(3A), L342 (1993)

4. Persaud, A. Analysis of slope-intercept plots for arrays of electron field emitters. *J. Appl. Phys.* **114**, 154301 (2013)

5. Gotoh, Y., Tsuji, H. & Ishikawa, J. Relationships among the physical parameters required to give a linear relation between slope and intercept of Fowler-Nordheim plots. *Ultramicroscopy* **89**, 63-67 (2001)

6. Xia, J., Chen, F., Li, J. & Tao, N. Measurement of the quantum capacitance of graphene. *Nat. Nanotechnol.* **4**, 505 (2009)

7. Robertson, J. High dielectric constant gate oxides for metal oxide Si transistors. *Reports Prog. Phys.* **69**, 2 (2006)

8. Jung, J. & Macdonald, A. H. Tight-binding model for graphene π-bands from maximally localized Wannier functions. *Phys. Rev. B - Condens. Matter Mater. Phys.* **87**, 195450 (2013)
